# Demographics and angiographic patterns in young and very young adults (≥35-40years of age) with coronary artery disease (CAD)

**DOI:** 10.1186/1749-8090-10-S1-A71

**Published:** 2015-12-16

**Authors:** Fuad Z Abdullayev, Rashad M Makhmudov, Imadaddin M Bagirov, Nigar J Kazimzade, Larisa S Shikhiyeva

**Affiliations:** 1 Dept Cardiac Surgery, Topchibashev Research Centre of Surgery, Baku, AZ 1122, AZ 1122, Azerbaijan; 2 Dept Cardio - Vascular Diseases, Central Hospital of Oil Workers, Baku, AZ 1025, Azerbaijan

## Background/Introduction

Significant differences in the risk predictors and coronary angiographic patterns between young (≤35-40 years of age) and older (>40 years) patients with CAD, cause different treatment strategies and outcomes among these groups.

## Aims/Objectives

To assess risk profile and coronary angiographic variables in young adults with stable angina (SA) and acute coronary syndrome (ACS).

## Method

Enrolled 70 patients 27-40years of age (38,5 ± 0,3) with CAD, including 9 (12,8%) - ≤35 years old. SA verified in 50 (71,4%) patients, among them 35(70%) with early MI; ACS - in 20(28,5%) patients, including 11(61%) with early MI. With regard to the coronary arteries (CA), attention was paid to the presence of any luminal narrowing, number of CA and segments involved.

## Results

Risk predictors presented with: early MI in 46 (65,7%) patients; smoking (>1 pack/day) in 26 (37,1%); family history of CAD in у 10 (14,3%); AH in 13 (18,5%); DM in 5 (7,1%); LVEF≥35-40% in 15 (21,4%); MV dysfunction (III-IV) in 5(7,1%); LV aneurysm in 6 (8,6%). BMI 25-30kg/m2 verified in 42,4% patients, BMI> 30кг/м2 - in 25,8%.

1VD revealed in 21 (30%) patients; 2VD - in 17 (24,3%); ≤3VD - in 32 (45,7%). RCA lesion verified in 39 (55,7%) patients; LAD - in 69 (98,6%); LCx- in 43 (61,4%); Left main-in in 4 (5,7%).

70 patients underwent CABG: 58 (82,9%) - On-pump, 12 (17,1%) - OPCAB with number of anastomoses 2,8 ± 0,1 & 1,0, accordingly. 9 (12,9%) patients underwent CABG on 5-48 months after early PCI (5 patients- on 9-12 months after PCI).

Risk predictors, coronary angiographic patterns, and in-hospital results of CABG compared in ACS and SA groups.

## Discussion/Conclusion

1. Young adults with ACS manifest with prevalence of patients ≥30-35 years, non atheromatous 1VD, and one independent risk predictor; 2. Young adults with SA dominate with patients 35-40years, atheromatous multi-VD, early MI, LV dysfunction, and ≤2-3 risk predictors; 3. Proportion of patients ≥30-35years in ACS and SA groups comprised 2:1.

